# Role of Iberin as an anti-apoptotic agent on renal ischemia-reperfusion injury in rats

**DOI:** 10.25122/jml-2022-0281

**Published:** 2023-06

**Authors:** Yahiya Ibrahim Yahiya, Najah Rayish Hadi, Ahmed Abu Raghif, Heider Qassam, Noor Ghaffar Said AL Habooby

**Affiliations:** 1 Deptartment of Pharmacology, Faculty of Pharmacy, University of Alkafeel, Najaf, Iraq; 2 Department of Pharmacology and Therapeutics, Faculty of Medicine, University of Kufa, Kufa, Iraq; 3 Deptartment of Pharmacology, College of Medicine, Al Nahrain University, Baghdad, Iraq; 4 Medical College, University of Kufa, Kufa, Iraq

**Keywords:** ischemia, IRI, rats, Iberin

## Abstract

Ischemia-reperfusion injury (IRI) is a major contributor to acute and chronic kidney failure, heart failure, and ischemic stroke. This study aimed to investigate the therapeutic potential of Iberin, known for its anti-inflammatory, antioxidant, and antiapoptotic properties, in a rat model of renal IRI. Twenty-four adult male rats were randomly divided into four groups: Group I (Sham group) underwent laparotomy without IRI induction; Group II (Control group) underwent laparotomy followed by renal artery clamping for 30 minutes to induce ischemia, followed by 2 hours of reperfusion; Group III (Iberin treatment group) received a pre-injection of Iberin (15 mg/kg) and underwent 30 minutes of ischemia followed by 2 hours of reperfusion; and Group IV (Vehicle-treated group) received the vehicle (ethanol) 1 hour prior to ischemia and reperfusion induction. Iberin was diluted with ethanol. Biomarkers associated with inflammation, oxidative stress, and apoptosis were measured using enzyme-linked immunosorbent assay. Iberin treatment significantly reduced levels of inflammatory cytokines interleukin-1β (IL-1β) and IL-6, Bcl-2-associated X protein (BAX), tumor necrosis factor α (TNF-α), nuclear factor kappa p56, high mobility group B1, and neutrophil gelatinase-associated lipocalin. Moreover, Iberin increased levels of heat shock protein and Bcl2 compared to the control and vehicle groups. Iberin treatment prolonged the ischemic tolerance of renal tissue, potentially preventing or delaying irreversible injuries. These findings highlight the potential of Iberin as a promising candidate for mitigating renal injury caused by ischemia-reperfusion, due to its ability to modulate inflammatory markers.

## INTRODUCTION

Renal ischemia-reperfusion injury (IRI) is a common form of intrinsic acute kidney injury that can occur in various clinical scenarios, including renal transplantation [1]. The pathogenesis of IRI involves complex molecular and biochemical changes that lead to inflammation, apoptosis, and oxidative stress, resulting in kidney and organ damage [2,3]. At the molecular level, IRI induces intracellular and cellular axis including transcriptional reprogramming, apoptosis, and cell death in addition to the activation of the innate and adaptive immune responses [4,5]. Extensive research has contributed to our understanding of the molecular mechanisms underlying ischemia-reperfusion injury, leading to the exploration of novel therapeutic strategies for managing inflammation associated with ischemia/reperfusion [6]. However, despite various pharmacological interventions aimed at reversing IRI, few have demonstrated significant clinical efficacy. The duration of ischemia during renal injury varies, ranging from 20 to 75 minutes, and for this study, we chose a standardized 30-minute duration, as it represents an average timeframe that induces severe changes in tubular epithelium, such as acute tubular necrosis [7-9]. Under the influence of ischemia, concurrent with an increase in the concentration of acute tubular necrosis (ATN), specific changes occur in the renal tubules. These changes include the smoothing of the tubular epithelium, tubular dilatation, and the formation of casts. The pronounced inflammatory reaction in IRI, driven by cytokines and chemokines, contributes to renal tissue damage [10]. In this study, we investigated the potential of Iberin in mitigating renal IRI using a rat model, aiming to explore its anti-inflammatory, antioxidant, and antiapoptotic properties.

## MATERIAL AND METHODS

### Animals

A total of 24 adult male rats, aged 2 months, with an average weight of 300±50 g, were used in this study. These animals were housed in the animal laboratory at the University of Kufa, Faculty of Sciences. All experimental procedures were conducted in accordance with the guidelines and regulations set by the panel committee for medical ethics at the University of Kufa.

### Study design

The rats were randomly assigned to four groups, with six animals in each group, as follows:

1. Sham group (negative control): Rats underwent anesthesia by intraperitoneal injection of 100 mg/kg ketamine and 10 mg/kg xylazine, followed by laparotomy without induction of renal ischemia-reperfusion injury (IRI).
2. Control group: Rats underwent bilateral renal ischemia for 30 minutes by vascular clamping of the renal arteries, followed by reperfusion for 2 hours.
3. Vehicle-treated group: Rats received an intraperitoneal injection of ethanol (the vehicle of Iberin) 1 hour prior to the induction of ischemia and reperfusion.
4. Iberin-treated group: Rats were intraperitoneally administered Iberin at a dose of 15 mg/kg, 1 hour before the induction of ischemia and reperfusion.

### Tissue homogenate preparation

The left kidneys of the rats were harvested and deep frozen at -80°C. The tissue samples were then thawed and washed several times with cold phosphate-buffered saline (PBS). Subsequently, the tissue samples were weighed and homogenized in a PBS solution (pH 7.4) at a ratio of 1:10 (weight of tissues/volume of lysis buffer). The homogenates were prepared using a solution containing a 1% protease inhibitor cocktail and 1% Triton X-100 [11]. The homogenates were then centrifuged at 3000 rotations per minute for 20 minutes at 40°C [12]. To avoid repeated freeze-thaw cycles, the supernatants were collected and divided into different aliquots. The aliquots were then deep frozen and used for ELISA measurements of inflammatory, oxidative, and apoptotic markers at the Bioassay Technology laboratory using the sandwich ELISA method.

### Statistical analysis

The data obtained from the study were analyzed using the Statistical Analysis System (SAS) version 9.1. One-way analysis of variance (ANOVA) was used to determine significant differences among the study groups. The Tukey post hoc test was used for pairwise comparisons.

## Results

### Effect of IRI and Iberin on Study Parameters

#### Neutrophil gelatinase-associated lipocalin (NGAL)

**Figure 1:**
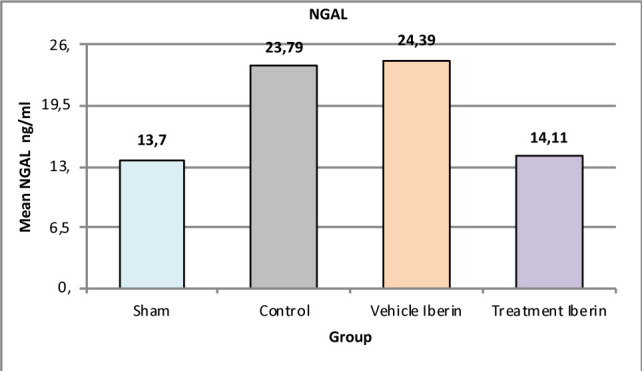
Mean tissue levels of NGAL across experimental groups

Compared to the sham group, the levels of NGAL were significantly increased in the control group and vehicle group (P<0.05), as shown in Figure 1. However, treatment with Iberin resulted in a considerable reduction in NGAL levels compared to the control and vehicle groups (p<0.05).

#### High mobility group B1 (HMGB1)

**Figure 2:**
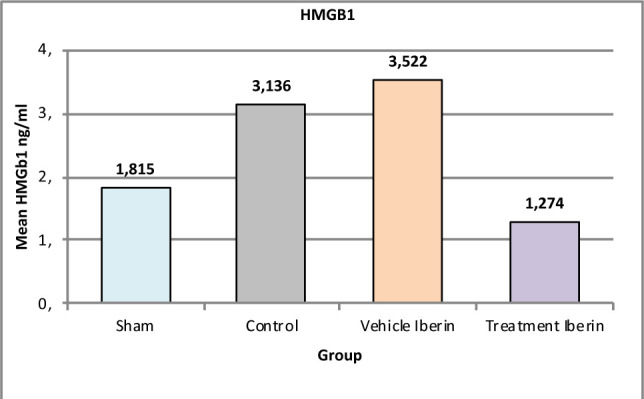
Mean tissue levels of HMGB1 across experimental groups

Mice subjected to renal IRI exhibited elevated levels of HMGB1 compared to the sham group (p<0.05), as shown in Figure 2. Treatment with Iberin resulted in a marked reduction in the levels of HMGB1 (p<0.05).

#### Nuclear factor kappa p65

**Figure 3:**
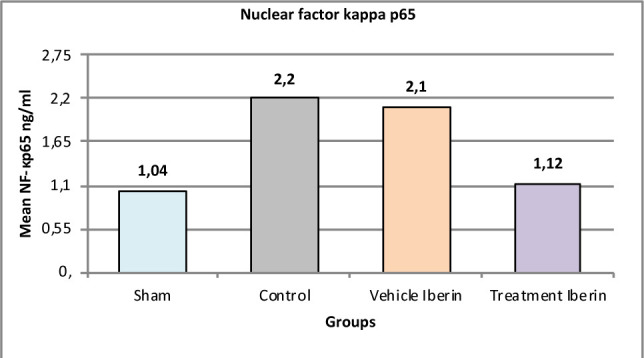
Mean tissue levels of nuclear factor kappa p65 across experimental groups

The mean levels of nuclear factor kappa p65 were significantly higher in the control group and vehicle group compared to the sham group (p<0.05), as shown in Figure 3. In contrast, treatment with Iberin led to a reduction in nuclear factor kappa p65 levels compared to the control group (p<0.05).

#### Interleukin-1β (IL-1β) and IL-6

**Figure 4:**
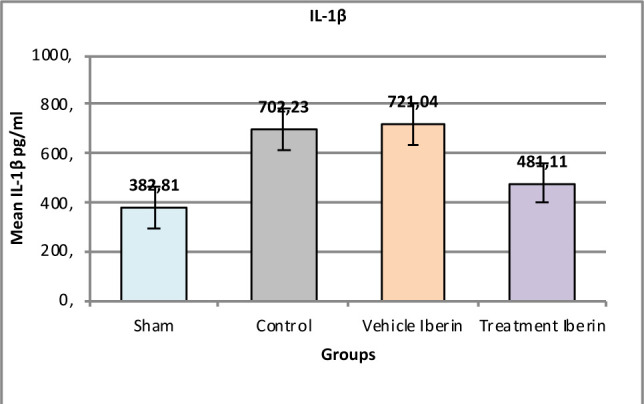
Mean tissue levels of IL-1β across experimental groups

**Figure 5:**
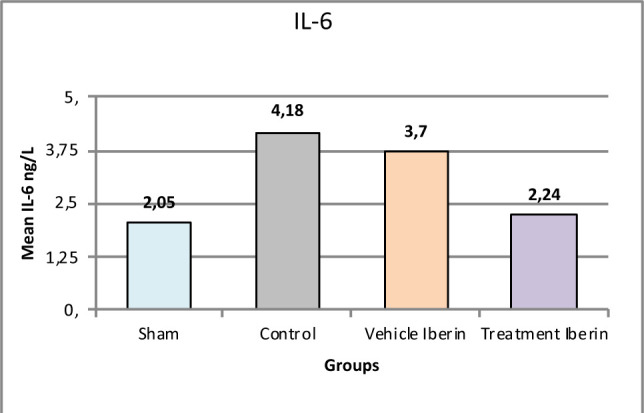
Mean tissue levels of IL-6 across experimental groups

The levels of IL-1β were significantly higher in the control group and vehicle group compared to the sham group (p<0.001) (Figure 4). These levels were significantly decreased in the Iberin-treated group (p<0.001). Similarly, the levels of IL-6 were significantly increased in the control group and vehicle group compared to the sham group (p<0.05) (Figure (5). Treatment with Iberin resulted in a significant reduction in IL-6 levels, approaching levels observed in the sham group (p<0.05).

#### Tumor necrosis factor-α (TNF-α)

**Figure 6:**
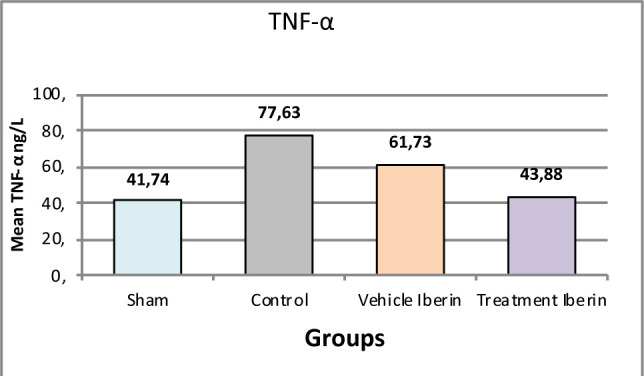
Mean tissue levels of TNF-α across experimental groups

The mean TNF-α levels were higher in the control group and vehicle group compared to the sham group (p<0.05). Treatment with Iberin significantly decreased the levels of TNF-α (p<0.05) (Figure 6).

### Effects of Renal IRI and Iberin on Apoptotic Mediators

#### Bcl-2-associated X protein (BAX)

**Figure 7:**
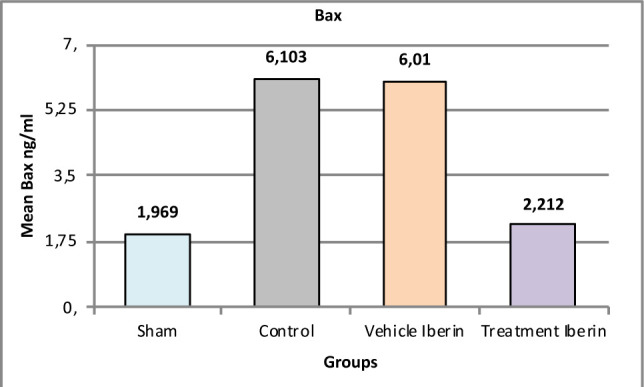
Mean tissue levels of BAX across experimental groups

The levels of BAX in the renal tissues were higher in the control group and vehicle group compared to the sham group (p<0.05). Iberin treatment resulted in a significant reduction in the levels of BAX in the kidney tissues (p<0.05) (Figure 7).

#### Heat shock protein 27 (HSP27)

**Figure 8:**
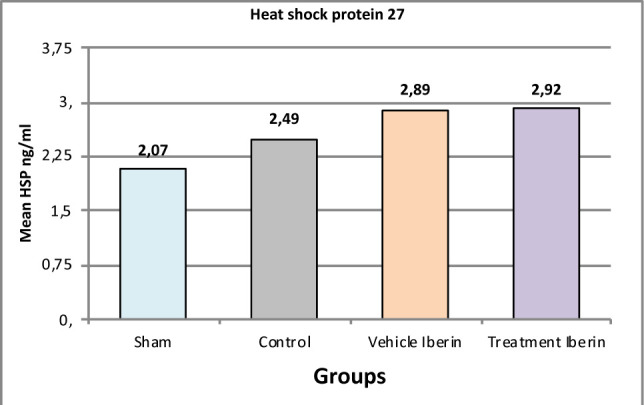
Mean tissue levels of HSP27 across experimental groups

The levels of HSP27 in kidney tissues were significantly higher in the Iberin-treated group compared to the sham group (p<0.05) (Figure 8).

#### B-cell lymphoma 2 (Bcl-2)

**Figure 9:**
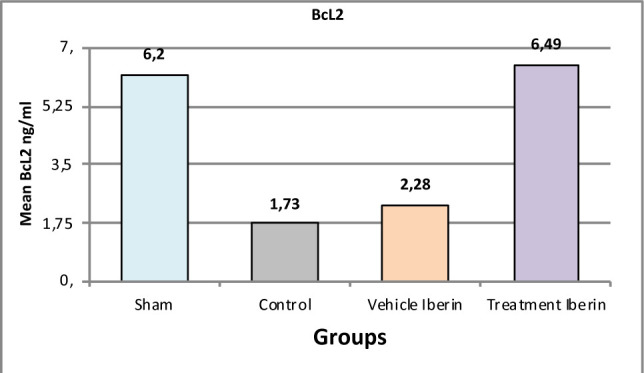
Mean tissue levels of TNF-α across experimental groups

Mice subjected to ischemia and reperfusion had significantly lower levels of the Bcl-2 compared to the sham group (p<0.05) (Figure 9). These levels were significantly elevated when treated with Iberin (p<0.05).

## DISCUSSION

The most effective treatment for reperfusion syndrome should be a drug that can prevent the formation of active oxygen radicals and prevent secondary damage to cell membranes and reperfusion syndrome. Moreover, treatment modalities can prolong the time that the tissue can withstand the ischemic effect and delay or prevent irreversible injuries [13,14]. In the complex therapy of severe conditions, some antioxidants should be used to neutralize reactive oxygen species (ROS), which significantly improve the results of treatment. Prevention of reperfusion syndrome eliminates the formation of active oxygen radicals, providing oxygen directly to the cell, restores the aerobic metabolic pathway, and increases its energy value, which helps protect tissues and organs from secondary damage [15].

### Effect of Iberin on IL-1β2, IL-6 and TNFα

In our study, the administration of Iberin resulted in a significant decrease in the levels of IL-1β and TNF-α compared to the control and vehicle groups after IRI. These findings are consistent with previous experimental studies that have demonstrated an increase in IL-1β and TNF-α levels after a period of ischemia followed by reperfusion in rats. Elevated levels of IL-1β and TNF-α have been associated with endothelial dysfunction and contribute to the pathogenesis of IRI [7,16]. Furthermore, in a model of renal ischemia, it has been observed that the TNF-α gene expression is highly elevated in injured kidney tissues following a nephrectomy of the right kidney and subsequent ischemia in the left kidney [17]. Moreover, other experimental studies conducted on rat models showed that the level of IL-1β was increased in injured renal tissues after 30 min ischemia and then 2hrs after reperfusion [18,19].

### Effect of Iberin on Bcl-2 and BAX

In our experimental study, pretreatment with Iberin significantly reduced the levels of BAX compared to the other groups. These findings contradict a previous study where the levels of BAX were substantially decreased in the untreated group compared to the sham group, while the level of Bcl-2 was higher in the untreated group than in the sham group after 30 minutes of renal ischemia followed by 72 hours of reperfusion [20,21].

### Effect of IRI and Iberin on heat shock proteins (HSP)

Wang *et al*. reported that HSP is a well-known molecule with antiapoptotic properties, as it interferes with various mechanisms involved in cell apoptosis. Induction of HSP by nonlethal insults, both in vitro and in vivo, is associated with acquired cytoprotection [22]. In this context, pretreatment with Iberin may play a crucial role in this mechanism. Other studies have also demonstrated that the mRNA expression of Bcl-2 and heat shock proteins, which are key factors for cell viability and apoptosis, undergo changes during IRI [23]. These findings suggest that there may be differential regulation of mRNA among different organs in response to IRI, leading to alterations in the function of genes involved in cell death [24].

### Effect of IRI and Iberin on HMGB1

HMGB1, through its binding to the receptor for advanced glycation end-products (RAGE), activates proinflammatory mechanisms that can contribute to tissue injury. Therefore, targeting HMGB1 could represent a novel approach to mitigate the effects of IRI [25]. By antagonizing HMGB1, the inflammatory response can be attenuated, potentially reducing tissue damage associated with IRI [26,27].

### Effect of IRI and Iberin on NF-κB

The observed reduction in nuclear factor kappa p56 (NF-κB) levels in each treatment group may be attributed to the potential inhibitory effect of these agents on NF-κB. Inhibition of the NF-κB pathway has been recognized for its potential role in protecting against ischemia-reperfusion injury (IRI), although the exact mechanism of action is not fully understood [28,29]. Activation of NF-κB has been shown to promote the expression of inflammatory genes, including IL-1β, IL-6, IL-10, and TNF-α, as well as adhesion factors [30,31]. The upregulation of adhesion molecules attracts more neutrophils and lymphocytes, leading to further injury to vascular endothelial cells. Therefore, the use of an anti-inflammatory agent could have a promising protective effect in renal IRI. These findings are consistent with the results of our study, where all three treatment modalities significantly reduced NF-κB levels [32].

### Effect of IRI, and Iberin on NGAL

NGAL, a well-established marker of kidney injury, exhibited a significant increase in the IRI group, consistent with previous studies [33]. However, in our study, treatment with Iberin resulted in a significant reduction in NGAL levels. This effect may be attributed to the anti-inflammatory and antioxidant properties of Iberin, which contribute to the protection of the kidney against IRI-induced injury.

## CONCLUSION

The research findings demonstrate that Iberin has a significant effect in lowering the levels of inflammatory markers compared to the other groups. Remarkably, the levels of these markers were nearly comparable to those of the sham group, indicating the potential anti-inflammatory properties of Iberin.
